# Experimental Investigation of Synchronous Grouting Material Prepared with Different Mineral Admixtures

**DOI:** 10.3390/ma15031260

**Published:** 2022-02-08

**Authors:** Jian-Fen Li, Yuan-Tao Liu, Shu-Jin Li, Yang Song

**Affiliations:** 1 School of Civil and Architectural Engineering, Changzhou Institute of Technology, Changzhou 213032, China; lijf@czu.cn (J.-F.L.); songy@czu.cn (Y.S.); 2 Department of Civil Engineering, Shanghai Jiaotong University, Shanghai 200240, China; liuyuantao2021@email.szu.edu.cn

**Keywords:** waste sediment, grouting materials, mineral admixtures, rheological properties

## Abstract

Waste sediment generated during tunnel construction is applied to prepare synchronous grouting material, where the influences of fly ash, slag powder, and bentonite on the rheological properties (such as consistency, fluidity, setting time, drainage rate, and stone rate) are studied. The results show that adding fly ash content increases the initial consistency, setting time, and fluidity of grouting material, but also increases its drainage rate and decreases its stone rate. The addition of slag powder results in a slight increase in the setting time and fluidity of the grouting material, yet a decrease in the initial consistency value. In contrast, with the addition of bentonite, both the initial consistency and fluidity of the grouting material decrease. Finally, the optimal mix ratio of high-performance and low-cost grouting materials is fixed to be 30% fly ash, 50% slag powder, and 10% bentonite. Therefore, the fluidity of grouting material can be 170 mm, with an initial consistency of 122 mm, setting time of 1050 min, stone rate of 96.2%, drainage rate of 1.5%, and 28-day compressive strength of 8.3 MPa.

## 1. Introduction

During tunnel construction, synchronous grouting is used to closely connect the stratum and segment lining to ensure the safety of the segment lining. Thus, high-flow synchronous grouting materials are increasingly and urgently needed [1]. With the development of grouting technology, many studies have focused on the development and application of new materials to meet new requirements in metro construction. Cement-based grouting material, which is made of cement, fly ash, bentonite, and other materials, is well researched and commonly used in tunnel construction [2,3].

Recently, Li, S. et al. [4] developed a quick-setting cement-based grouting material by incorporating polymer chemical materials. To solve the sand and grouting penetration, Li, L. et al. [5] developed a polymer grouting material that can effectively strengthen loose sand. Zhang, H. et al. [6] analyzed the influence of grouting material on the properties of the slurry and measured the strength, fluidity, workability, density, and drainage rate of the grouting at different ages. Consequently, the ratio of slurry materials is optimized. Xu, J. et al. [7] explored the influence of the composition, content of sand, and plasticity index of the shield tunnel on the performance of grouting material. They found that shield waste sediment can completely replace the bentonite in traditional synchronous grouting material. Additionally, the mechanism of each component on the performance of the slurry was analyzed and the ordinary shield mud sand grouting material was proposed. Wang, S. et al. [8] developed a two-liquid plastic shield tunnel synchronous grouting material composed of fly ash, cement, water glass series, and bentonite, and analyzed the workability, stability, gel properties, and strength of each component of the grouting material in relation to the slurry. Ding, Q. et al. [9] used industrial waste slag (slag, steel slag, and fly ash) and water glass to prepare a double liquid grouting material with a different gel time (3~300 s), high consolidation strength (0.1~25 MPa), and stone rate up to 100%. The grouting materials can regenerate industrial waste residues and reduce the consumption of grouting cement, providing the option of “green” and high-performance grouting materials. To solve the problem of poor corrosion resistance and volume shrinkage of traditional grouting materials, Wang, J. et al. [10] proposed a new type of alkali-activated geopolymer two-liquid grouting material. The shrinkage rate of the alkali-activated geopolymer slurry consolidation body increases with the content of metakaolin [11].

Reschke and Noppenberger [12] summarized the application of double grouting in the construction of the super-large diameter shield tunnel of the Brisbane Airport Link Road in Australia, and proposed that the early strength of the double grouting material should be developed quickly in situ. Sharghi [13] developed a new kind of two-liquid grouting material and measured its compressive strength, elastic modulus, and Poisson’s ratio. Many scholars [14,15,16] have studied the influence of SiO_2_ and CaCO_3_ on the performance of SAC (Sulpho Aluminate Cement). The results show that CaCO_3_ accelerates the hydration process of cement and shortens the setting time of the slurry. This is because Ca^2+^ reduces the conversion of Aft (Ettringite) to AFm (Monosulfate) in the system and enhances the SAC strength and impermeability. Yoon, J. and Chadi [17] studied the effect of fly ash on the performance of SAC. Their results showed that fly ash has little effect on the hydration of SAC, but the addition of fly ash increases the fluidity of the slurry and decreases the strength.

The existing shield synchronous grouting materials are mainly single-liquid cement slurry and cement–water glass slurry. The proportions of these slurries are only optimized when based on a traditional cement base. However, in tunnel construction, plenty of sediment is broken after the grinding and cutting of the multi-layer cutter head of the shield machine these sediments will damage the environment after improper handling. The existing sediment treatment is mainly used for the designated spoil ground or backfill. The preparation of synchronous grouting materials is a feasible way of utilizing waste sediment resources, which can not only reduce environmental pollution caused by sediment discharge and waste of manpower, material resources, and materials, but also reduce the cost of synchronous grouting materials [18,19,20].

In this study, sediment samples from Metro Line 2 of Changzhou City, Jiangsu Province, China are used and their basic physical and chemical properties (such as chemical composition, liquid plastic limit, and sand content) are analyzed. Then, the influences of fly ash, slag powder, and bentonite content on consistency, fluidity, and water dispersion performance resistance are studied, which provides key technological and theoretical support for the recycling of waste sediment in tunnel construction.

## 2. Materials and Methods

### 2.1. Raw Materials

**Figure 1:**
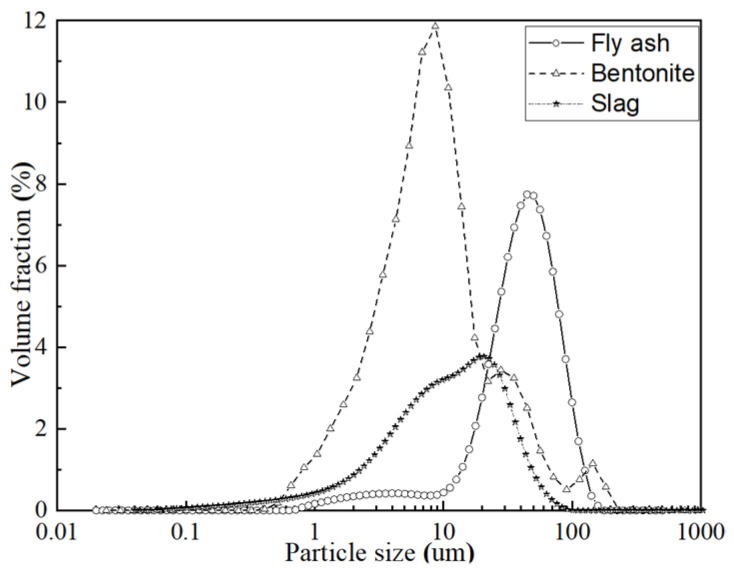
Particle size distribution of materials.

**Figure 2:**
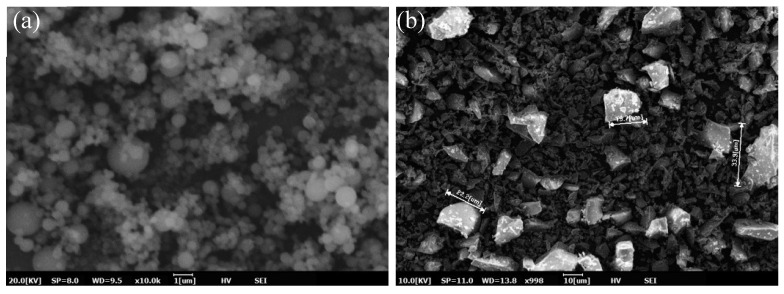
SEM images of raw materials: (**a**) fly ash and (**b**) slag.

In this study, grade 42.5 ordinary portland cement (P.O 42.5) produced by Conch Group Co., Ltd. in Anhui Province, China, with a specific surface area of 355 m^2^/kg, was used. Low calcium fly ash (class F) was produced by Jiangsu Huadian Wujiang Thermal Power Co., Ltd. in Shanghai City; slag powder (S95 slag powder) was produced by Shanghai Baogang Steel Group. Sodium bentonite with a fineness of 200 mesh, gum value of more than 4 mL/g, and expansion coefficient of more than 5 mL/g was used. The details of the main components above are shown in Table 1. The main contents of fly ash are Al_2_O_3_, SiO_2_ and Fe_2_O_3_ (81.8%). Slag mainly consists of CaO, SiO_2_, and Al_2_O_3_ (89.9%). The particle size distribution of raw materials is provided in Figure 1. The particle sizes of bentonite were concentrated in the range of 5–20 µm. Slag and fly ash consisted of relatively larger-sized particles as compared to bentonite. The SEM (Scanning Electron Microscopy) images of fly ash and slag are provided in Figure 2. It can be seen that fly ash particles are mainly spherical in shape, whereas slag consists of angular particles.

### 2.2. Waste Sediment

The waste sediment was produced by the shield construction of Metro Line 2 of Changzhou City, Jiangsu Province, China. Centrifugal dewatering showed that the sediment had 19.6% water content and 55% sediment concentration. The pH value of the waste sediment was 6.87 and the density was 2.67 kg/m^3^.

The waste sediment belonged to muddy, silty clay with a plastic limit, liquid limit, and plasticity index values of 23.3, 41.6, and 18.3, respectively. The main components of the waste sediment are shown in Table 1, and the particle size distribution of the waste sediment measured after drying is shown in Table 2.

### 2.3. Preparation of Samples

The weighed raw materials were mixed in a dry state and slowly stirred for 3 min. Then, water is added and the mixture was quickly and evenly stirred for 4 min. After being stirred, the fluidity, consistency, consistency loss, stone rate, drainage rate, and setting time of the fresh materials were tested, respectively. The model samples were formed in muddles and cured under the standard conditions of 20 ± 2 °C and RH 95 ± 2%. Finally, the compressive strength was tested on days 3 and 28.

The mix proportions of waste sediment grouting materials are shown in Table 3. The effects of fly ash, slag powder, and bentonite on the properties of waste sediment grouting materials were studied. Note that the solid is equal to the total amount of cement, fly ash, and slag powder, while the bentonite content is the sum of cement, fly ash, and slag powder.

### 2.4. Property Test

The fluidity of the grouting materials was tested according to GB/T 50448, “Technical specification for application of cement-based grouting materials”. The test was conducted according to the specification of the truncated cone fluidity test. The height of the truncated cone was 60 mm, while the top and bottom diameters were 70 mm and 100 mm, respectively. The grouting materials were filled into the cone which was situated on the glass pan (500 mm × 500 mm) and then the cone was vertically lifted without vibrations. The spread of the grouting materials was measured and reported in terms of fluidity at different intervals up to 2.5 h. The fluidity of the grouting materials was tested after every 30 minutes. The flow diameter was recorded in two perpendicular directions and the average value was reported.

Setting time, consistency, and consistency loss of the grouting materials were tested according to JGJ/T 70, “Test method for basic properties of building mortar”. The setting time was determined by a penetration resistance method using the ZKS-100 Mortar Consistency Meter manufactured by Shanghai Meiyu Instrument Equipment Co., Ltd. The length of the penetration needle was 25 mm and its penetration area was 30 mm^2^. The initial setting was reached when the penetration resistance of the needle was 0.3 MPa, and the final setting time was reached when the penetration resistance was 0.7 MPa. The consistency of the grouting materials was calculated by determining the sinking degree of the hammer falling into the grouting material. The results were calculated by taking the average up to an accuracy of 1 mm.

The stone rate and drainage rate of the grouting materials were tested according to the test requirements of T/CECS 563-2018, “Technical specification for application of synchronous grouting materials in shield tunnel”. The test was conducted in a cylinder with a volume of 250 mL. The cylinder was placed on a level surface and filled with fresh slurry, and an initial reading ($a_{0}$) was recorded. The drainage rate was calculated by measuring the scale corresponding to bleeding surface ($a_{1}$) and slurry surface ($a_{2}$) at 3 hours. The stone rate was calculated by measuring the scale corresponding to hardened slurry ($a_{3}$) after 3 days. The test was conducted at a temperature of 20 ± 2 °C, and at a relative humidity of greater than 50%.

(1) $$\mathrm{Drainage}\;\mathrm{rate}=\frac{a_{1}-a_{2}}{a_{0}}\times 100$$

(2) $$\mathrm{Stone}\;\mathrm{rate}=\frac{a_{3}}{a_{0}}\times 100$$

The compressive strength of the grouting material was determined according to the provisions of JGJ/T 70, “Test method for basic properties of building mortar”. The size of the sample was 70.7 mm × 70.7 mm × 70.7 mm, and the sample was loaded continuously and evenly at a rate of 0.05 kN/s. To ensure the accuracy of the test data, parallel experiments were carried out for each group: three sets of the consistency, fluidity, setting time, drainage rate, and stone rate tests were conducted for each group, and then average values were obtained. For the compressive strength test, at least twelve samples (four triplet models with the shape of 70.7 mm × 70.7 mm × 70.7 mm) were prepared for each mix proportion.

The morphology of hydration products was observed by the SEM machine (EM-30 Plus, COXEM Company, Daejeon, Korea). After the measurement of 28-day compressive strength, the fractured pastes were collected and put into a bottle containing isopropyl alcohol, aiming to terminate the hydration process of the broken samples. Afterwards, the samples were dried in an oven, polished using a digital ion coater with a thin layer of gold coating, and tested in the SEM machine.

## 3. Results and Discussion

### 3.1. Fresh Grouting Materials

**Figure 3:**
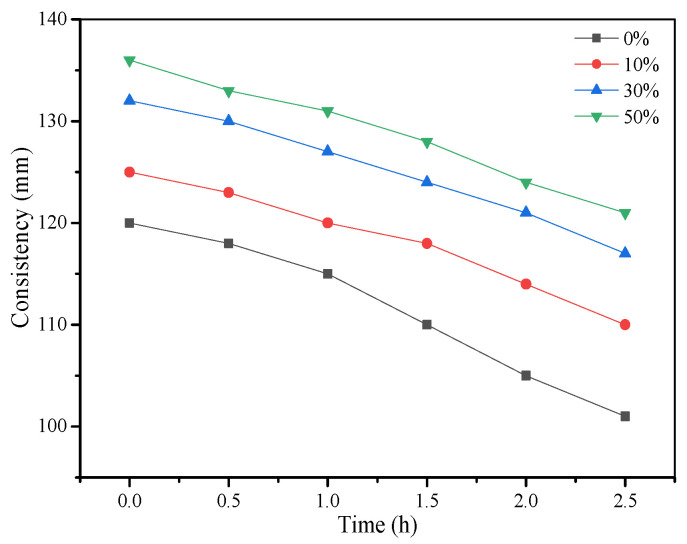
Influence of fly ash on consistency.

**Figure 4:**
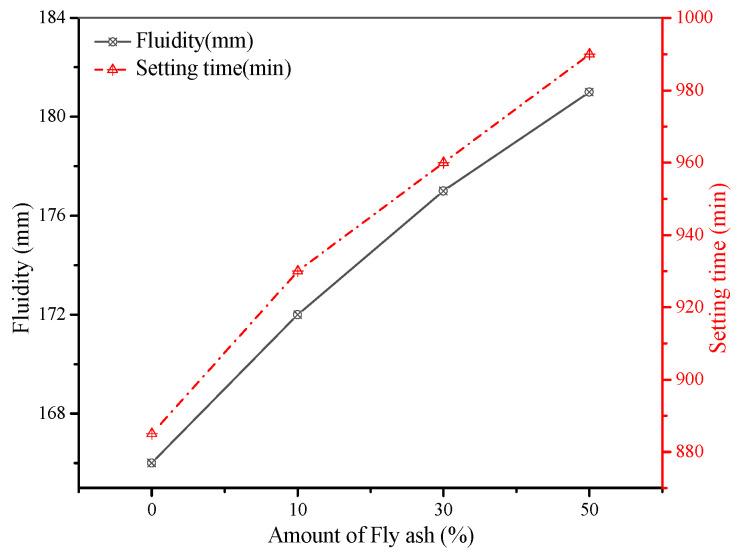
Influence of fly ash on fluidity and setting time.

The influence of fly ash content on the consistency of the fresh grouting material is shown in Figure 3. It can be seen that consistency of grouts has a direct relationship with the amount of fly ash. The consistency gradually increased from 120 mm to 136 mm when the amount of fly ash increased from 0% to 50%. Meanwhile, the consistency loss of each group was less than 10 mm/h. Similarly, the fluidity of the grouting materials also increased from 166 mm to 181 mm as fly ash content increased (Figure 4). The increase in the flow properties of cementitious materials due to the addition of fly ash particles has been reported in the literature [21,22,23] and is associated with the particle shape of fly ash, as shown in Figure 2a. It can be seen that fly ash consists of sphere-shaped particles, which can reduce inter-particle friction and hence increase the consistency and fluidity of fresh grouts. The influence of fly ash content on the setting of fresh grouts is presented in Figure 4. The setting time of grouts was prolonged due to the addition of fly ash. This phenomenon is related to the slow pozzolanic reaction of fly ash as compared to traditional cement. Since the consistency and fluidity are directly related to the pumpability of grouting materials, adding fly ash can improve the pumpability of grouting materials [24].

The stone ratio is calculated by taking the percentage of volume of grout concentration bodies to original grouts. A higher stone ratio means a higher stability of grouts. To ensure the quality of grouting construction, it is essential that the stone rate of slurry is more than 95% and the drainage rate is less than 3.5%. As shown in Table 4, due to the low reactivity of fly ash, the stability of fresh material will be reduced by adding fly ash, resulting in the increase in drainage rate and the decrease in stone rate. When the content of fly ash is 50%, the stone rate is 94.2%, and the drainage rate is 2.2%, which cannot meet the property requirements of grouting materials. Therefore, the optimum fly ash content should be 30%, where the stone rate is more than 95% and drainage rate is lower than 3.5%.

**Figure 5:**
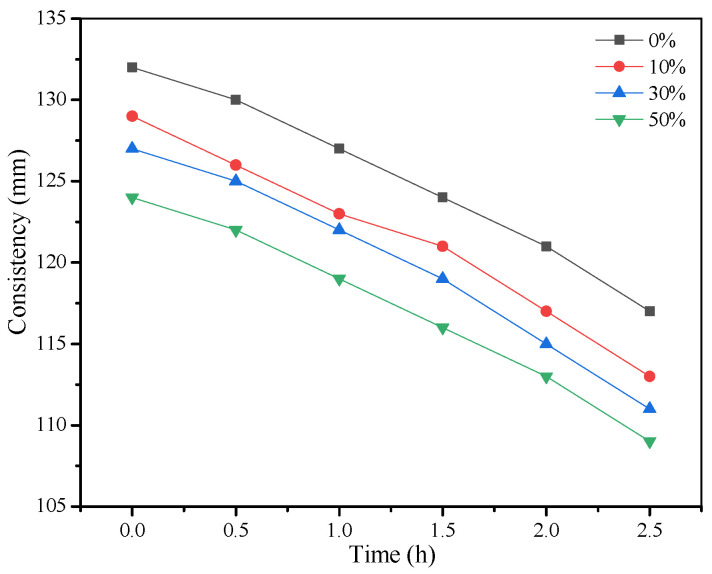
Influence of slag powder on consistency.

**Figure 6:**
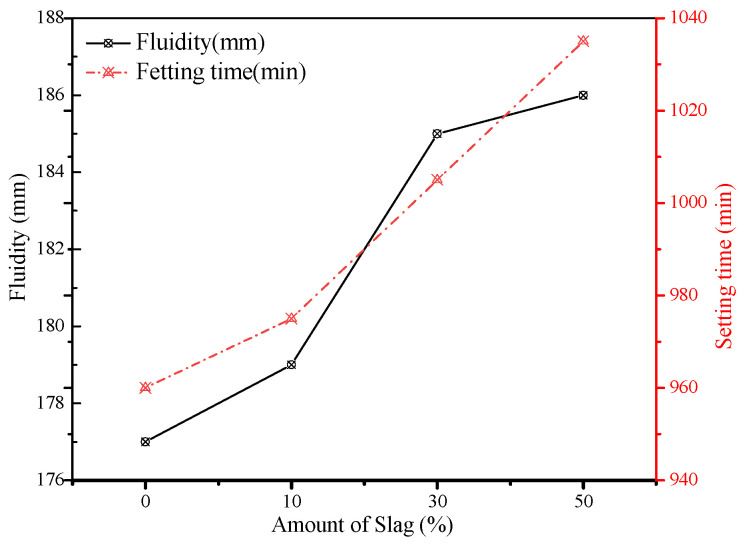
Influence of slag powder on fluidity and setting time.

After determining the optimum content of fly ash (30%) to achieve the desirable properties of grouting materials, the influence of slag powder on the properties of fresh grouting materials was studied. It should be noted that cement was gradually replaced by slag powder by 10%, 30%, and 50%. The results of consistency, fluidity, and setting time are presented in Figure 5 and Figure 6. The influence of slag powder on the consistency of the grouting material was opposite to that of fly ash as its addition slightly reduced the consistency of fresh pastes. The initial consistency of fresh pastes was reduced from 132 mm to 124 mm. However, the fluidity of the pastes was increased gradually. As observed from the SEM image of slag powder particles, they contain angular particles but with lower roughness (smooth surface morphology) as compared to cement particles, which can reduce the yield stress of fresh grouting material and gradually improves the fluidity of the mixture as cement is replaced by slag. However, its water-reducing capacity is lower than that of fly ash, which is mainly composed of sphere-shaped particles [25,26].

Due to the high viscosity between slag powder particles, the addition of slag powder will cause the slurry to be thicker and the consistency will be reduced [27]. When the content of slag powder increases from 0% to 50%, the consistency loss of each group can be kept within 10 mm/h, and the fluidity changed from 177 mm to 186 mm. The setting time of the grouts also increased as cement was replaced by the slag powder. Generally, slag also has high hydraulicity when mixed with cement and alkaline materials. In this study, cement was replaced by slag, which itself tends to show a higher degree of hydraulicity as compared to slag powder. Therefore, the heat of hydration generated during the reaction was slower when the amount of slag powder was increased, which increased the setting time of fresh pastes, as shown in Figure 4.

As seen in Table 4, the stability of the mixture is slightly reduced by adding slag powder, which is because the activity of slag powder is higher than that of fly ash, although it is still lower than that of cement. The cementitious property of the material will be decreased by replacing cement with slag powder. Because of the poor water-holding capacity of slag powder, the drainage rate will increase. When the content of slag powder is 50%, the stone rate and drainage rate are 93.6% and 2.4%, respectively. This cannot meet the performance requirements of grouting materials. Therefore, it was necessary to add bentonite to further optimize the rheological properties of the fresh pastes.

To study the influence of bentonite on the properties of the fresh grouting material, the proportions of cement, fly ash, and slag powder (20:30:50) were kept constant and bentonite content was changed from 0% to 15% with an interval of 5%. Compared with slag powder and fly ash, bentonite is a kind of claystone in nature, which has strong hygroscopicity and expansibility, and can be dispersed into a gelled and suspended state in water. Since the medium solution of bentonite has a certain viscosity, thixotropy, and lubricity, it is usually used in synchronous grouting to prepare suspension slurry with good rheological properties [28].

**Figure 7:**
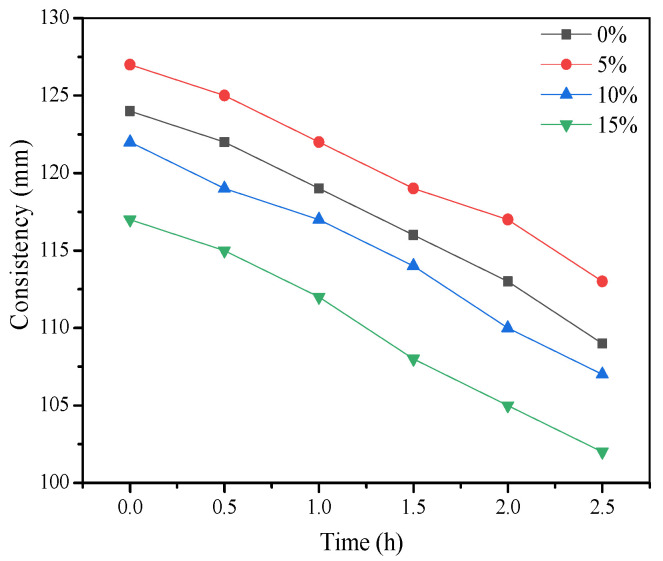
Influence of bentonite on consistency.

**Figure 8:**
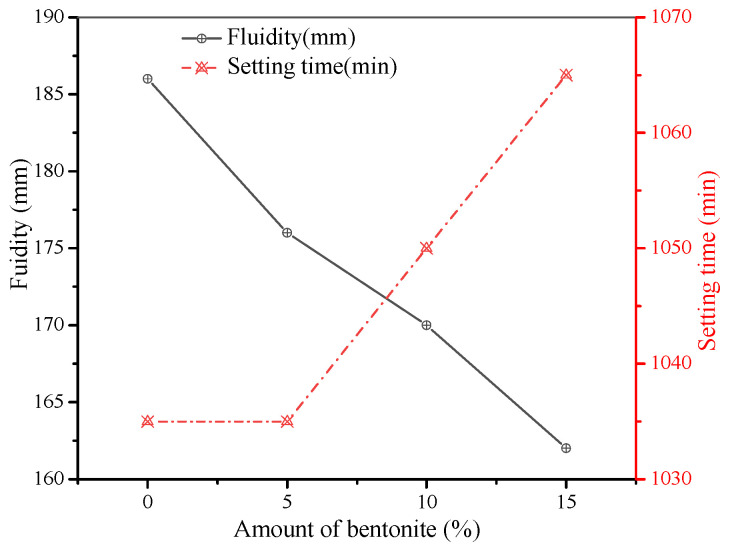
Influence of bentonite on fluidity and setting time.

As shown in Figure 7 and Figure 8, the initial consistency and fluidity of the grouting materials decreased with the increase in bentonite content from 0% to 15%. The consistency of the grouts was slightly decreased from 123 mm to 117 mm, while the fluidity was decreased from 186 mm to 162 mm. Meanwhile, the setting time was slightly prolonged but stayed in the range of 10~20 h. The stability and homogeneity of the material improved after adding bentonite as shown in Table 4. All the grouts containing 5%, 10%, and 15% bentonite in the grouting slurry met the requirements of synchronous grouting materials in the specification. When 10% bentonite was added, the drainage rate and stone rate were 1.6% and 96.2%, respectively.

Therefore, the use of fly ash and slag powder in the preparation of grouting materials can help to reduce the amount (which can also reduce cement-related carbon emission) and cost of cement (due to replacement of cement) and optimize the working properties of grouting material. Bentonite can further improve the stability of grouting materials by modifying their thixotropic properties, thus helping to achieve the desirable properties (suitable fluidity, consistency, setting time, stone rate, and drainage rate) required for practical application. Finally, the optimal mix ratio of grouting material is 30% fly ash, 50% slag powder, and 10% bentonite.

### 3.2. Hardened Grouting Materials

**Figure 9:**
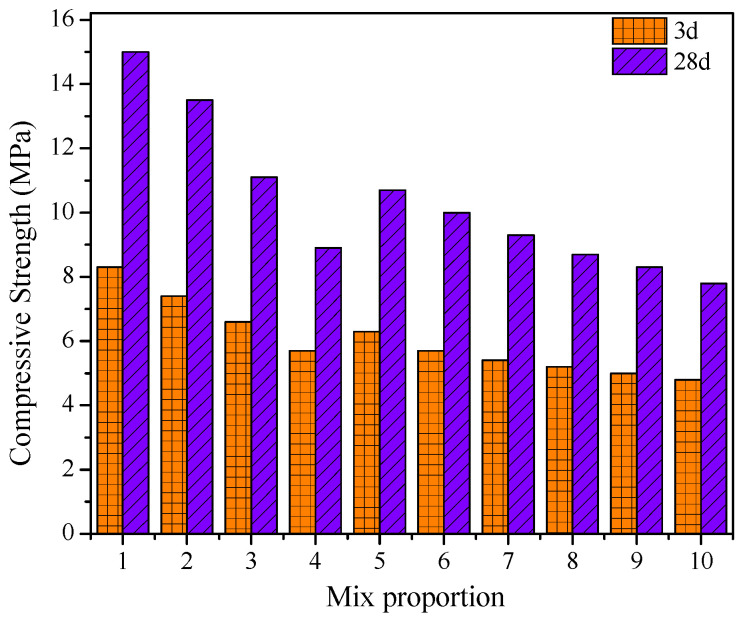
Compressive strength of grouting materials at different mix proportions.

Figure 9 shows the compressive strength of each group. Among them, the strength of group 1 without any mineral admixtures was the highest; the compressive strength at 3 days and 28 days was 8.3 MPa and 15.0 MPa, respectively.

With the addition of fly ash, slag powder, and bentonite, the early-age strength decreased gradually. Since fly ash is a kind of pozzolanic admixture and has lower cementitious activity than cement, the 28-day strength of group 4 with 50% fly ash decreased sharply from 15.0 MPa to 8.9 MPa. Slag powder has potential hydraulic properties, but its activity is still lower than that of cement, which also leads to a slight decrease in strength. The compressive pressure of groups 7–10 containing 30% fly ash and 50% slag powder was only around 9.3 MPa at 28 days. Meanwhile, bentonite is an excellent admixture to adjust the rheological properties, yet still leads to decreased mechanical properties of the slurry.

For the optimum mix proportion of 30% fly ash, 50% slag powder, and 10% bentonite, the compressive strength values at 3 days and 28 days were 5.0 MPa and 8.3 MPa, respectively. This fully meets the mechanical property requirements in synchronous grouting.

### 3.3. Microstructure of Hardened Grouting Materials

**Figure 10:**
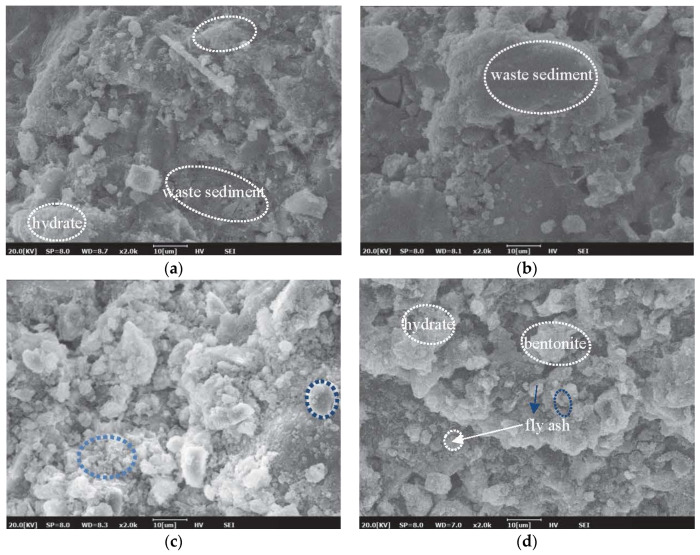
SEM of different samples cured for 28 days: (**a**) sample 1; (**b**) sample 6; (**c**) sample 7; (**d**) sample 9.

Figure 10 shows the microstructure of the hardened paste of each experimental group, where the influence of different mineral admixtures on the properties of grouting materials can be compared and analyzed. Due to the addition of fly ash, spherical particles can be observed in the structure. Hydration products have a higher flocculent structure when fly ash and slag are absent, as observed in Figure 10a. With the addition of fly ash and slag powder, the flocculent and acicular hydration products decrease, which leads to the increase in unreacted and low cementitious particles in the structure (Figure 10). The presence of hydration products surrounding waste sediment can observed in Figure 10b, which shows the possibility of reaction of waste sediment with the solid binder containing cement, fly ash, and slag. When the amount of slag is increased from 30% to 50%, a large number of reacted slag particles can be observed, as shown in Figure 10c. Meanwhile, with the addition of bentonite, the microstructure becomes dense, which is due to the water-swelling characteristics of bentonite. This can significantly improve the impermeability of the material [28,29].

## 4. Conclusions

(1) With the addition of fly ash, the initial consistency, setting time, and fluidity of grouting materials increase. However, due to the low activity of fly ash, the stability of the material will be reduced, resulting in the increase in drainage rate and the decrease in stone rate. In the experiment, the optimum content of fly ash was 30%.

(2) The addition of slag powder can slightly increase the setting time and fluidity of grouting materials but can decrease the initial consistency. Considering the relationship between material properties and cost, the optimal slag content is 50%.

(3) Bentonite has excellent hygroscopic and expansive properties, which can turn grouting materials into a homogeneous suspension slurry. With the addition of bentonite, the initial consistency and fluidity of grouting materials decreased, and 10% bentonite is optimal.

(4) The admixture of the three minerals can significantly control the rheological properties of waste sediment grouting materials. The optimal mix ratio is 30% fly ash, 50% slag powder, and 10% bentonite, to prepare high-performance, low-cost grouting materials with a fluidity of 170 mm, initial consistency of 122 mm, setting time of 1050 min, stone rate of 96.2%, drainage rate of 1.5%, and 28-day compressive strength of 8.3 MPa.

## Figures and Tables

**Table 1:** The main components of raw materials and waste sediment.

Name	MgO	CaO	Al_2_O_3_	Fe_2_O_3_	SiO_2_	Na_2_O	K_2_O	SO_3_	Others
Cement	1.1	64.4	4.1	4.6	21.6	0.1	0.6	2.8	0.69
Fly ash	1.8	6.8	23.5	8.9	49.4	0.3	1.3	1.4	6.6
Slag powder	5.9	41.6	12.6	0.4	35.7	0.2	0.3	1.7	1.6
Waste sediment	67.0	1.4	14.3	0.4	3.4	2.0	0.9	1.2	9.4

**Table 2:** Particle size distribution of the waste sediment.

Particle Size/mm	<0.005	0.005~0.05	0.05~0.10	0.10~0.25	>0.25
Ratio/%	7.6	9.4	47.5	33.7	1.8

**Table 3:** The mix proportions of waste sediment grouting materials.

No.	Water Solid Ratio	Sand Binder Ratio	Cement:Fly Ash:Slag Powder	Bentonite/%
1	0.45	1:1	100:0:0	0
2	0.45	1:1	90:10:0	0
3	0.45	1:1	70:30:0	0
4	0.47	1:1	50:50:0	0
5	0.45	1:1	60:30:10	0
6	0.45	1:1	40:30:30	0
7	0.45	1:1	20:30:50	0
8	0.45	1:1	20:30:50	5
9	0.45	1:1	20:30:50	10
10	0.45	1:1	20:30:50	15

**Table 4:** Drainage rate and stone rate of grouting materials with different mix proportions.

Sample	1	2	3	4	5	6	7	8	9	10
Drainage rate/%	1.1	1.3	1.7	2.2	1.8	2.1	2.4	1.9	1.5	1.2
Stone rate/%	97.2	96.8	95.3	94.2	95.1	94.7	93.6	95.0	96.2	97.0

## Data Availability

The data presented in this study are available upon request from the corresponding author.
